# Study on molecular mechanisms of nattokinase in pharmacological action based on label‐free liquid chromatography–tandem mass spectrometry

**DOI:** 10.1002/fsn3.1157

**Published:** 2019-08-22

**Authors:** Xia Pan, Pengyu Liang, Luyao Teng, Yuhao Ren, Jixian Peng, Weizhi Liu, Yan Yang

**Affiliations:** ^1^ Key Laboratory of Marine Genetics and Breeding, College of Marine Life Sciences Ocean University of China Qingdao China; ^2^ Shandong Ruiying Pioneer Pharmaceutical Co., Ltd Heze China; ^3^ Laboratory for Marine Biology and Biotechnology Qingdao National Laboratory for Marine Science and Technology Qingdao China

**Keywords:** cardiovascular disease, disease prevention, label free, nattokinase

## Abstract

Cardiovascular diseases (CVDs) are the leading causes of premature death and disability in people around the world. Therefore, the prevention and treatment of CVDs has become an important subject. In this study, we verified the thrombolytic activities of a nattokinase‐like protease named NK‐01 in vivo. Label‐free liquid chromatography–tandem mass spectrometry (LC‐MS/MS) technique was used in our study. NK‐01 could inhibit the activity of coagulation factors though the up‐regulation of proteinase C inhibitors and protein S. NK‐01 also could inhibit the angiotensinogen conversion to AngII and promote the degradation of kininogen to reduce the blood pressure. In addition, NK‐01 could increase the content of paraoxonase 1, which could prevent atherosclerosis. In our study, we found that NK‐01 cloud effect some key proteins which participant in CVDs associated metabolic processes such as coagulation system, blood pressure, and atherosclerosis. Taken together, the underlying molecular mechanisms for the biological beneficial of NK‐01 were investigated. Our proteomic study will provide further theoretical basis for application of NK in prevention or adjuvant treatment in biomedicine areas.

## 1 INTRODUCTION

WHO reported that 17.7 million people died of cardiovascular diseases (CVDs) every year and that counts for 31% of all death in the world (McAloon et al., 2016). Although the formation of CVDs is a complex pathological process, it frequently occurs suddenly and leads to disability or death (World Health Organization, 2013). For example, atherosclerosis is the underlying pathology of CVDs. The process of atherosclerosis is the deposition of fat and cholesterol in the lumen of blood vessels, which results in the reduced elasticity of blood vessels and the formation of thrombus. Thrombus can easily cause coronary heart disease and stroke (Mendis et al., 2011).

Some effective ways had been taken to reduce the incidence and mortality of CVDs. The primary preventions of CVDs are healthy diet, regular physical activities, and prohibition of tobacco products. For secondary prevention, pharmacologic interventions are necessary. The data from clinical trials have proved that medicines, like aspirin, statins, and blood pressure medicines, played important roles in managing CVDs (Baigent et al., 2009, 2010; Law, Morris, & Wald, 2009). However, CVD is a chronic disease that requires long‐term medical treatment. The patients usually have poor adherence to drugs, and the benefits of medications cannot be fully realized (Haynes, McKibbon, & Kanani, 1996; O'Flaherty, Buchan, & Capewell, 2013). In addition, most drugs have some side effects (Beltowski, Wojcicka, & Jamroz‐Wisniewska, 2009; Hankey & Eikelboom, 2006; Klegerman, 2017; Toh et al., 2012). For example, thrombolytic agents could lead to the unwanted internal bleeding and their half‐lives are short in vivo Nordt & Bode, 2003). Statins could lower the plasma low‐density lipoprotein (LDL) cholesterol, and it could also cause sympathy and severe rhabdomyolysis (Beltowski et al., 2009). As an antiplatelet drug, aspirin plays an important role in the prevention of CVDs. But the curative effects of aspirin vary from person to person and some people showed poor antiplatelet effect, which was called “aspirin resistance” (Georgiadis et al., 2013).

Nattokinase is a kind of serine protease with strong thrombolytic activity (Sumi, Hamada, Tsushima, Mihara, & Muraki, 1987). It was extracted from fermented beans such as natto, douche, and tempeh and can be absorbed through the intestine (Fujita, Hong, et al., 1995). Nattokinase was widely studied as a dietary supplement and nutritional food, which has potential to prevent and treat CVDs (Dabbagh et al., 2014; Weng, Yao, Sparks, & Wang, 2017). Hongjie Chen et al. reported that nattokinase was a promising alternative in prevention and treatment of cardiovascular diseases by possessing a variety of favorable cardiovascular effects, such as fibrinolytic activity, antihypertensive, antiatherosclerotic, and lipid‐lowering, antiplatelet, and neuroprotective effects (Chen et al., 2018). The consumption of natto has been linked to a reduction in CVD mortality. Compared with other thrombolytic drugs, nattokinase has higher thrombolytic activity, which was about 4× higher compared with urokinase (Fujita, Hong, et al., 1995). Nattokinase is more sensitive to cross‐linked fibrin than fibrinogen, which could effectively prevent internal bleeding (Fujita, Ito, Hong, & Nishimuro, 1995). Ji Young Kim et al. found that nattokinase resulted in a reduction in systolic blood pressure (SBP) and diastolic blood pressure (DBP) of hypertensive patients (Kim et al., 2008). Ren nina et al. had proved that nattokinase also played an important role in preventing atherosclerosis of patients (N. N. Ren, Chen, Li, Mcgowan, & Lin, 2017).

Recently, we found a nattokinase‐like protease named NK‐01, which shared 99% sequence identity with the nattokinase (Genebank number: AHZ12722.1) and composed of multiple fragments. Through casein plate method, we showed that NK‐01 has better fibrinolytic activity than urokinase (Y. Ren, Pan, Lyu, & Liu, 2018). In this study, we confirmed the in vivo thrombolytic activity of NK‐01. Based on label‐free technology, we studied the effects of NK‐01 on the proteomic profiling of plasma proteins in rats and further analyzed the possible mechanisms of its biological functions in the prevention of cardiovascular diseases.

## 2 MATERIALS AND METHODS

### 2.1 Chemicals

Nattokinase NK‐01 was prepared in our laboratory. Urokinase was obtained from Nanda Co., Ltd, Pelltobarbitalum Natricum was purchased from Solarbio. Trypsin was purchased from Promega, and iodoacetamide was purchased from Sigma. MeOH, formic acid, CAN, and TCEP were purchased from ThermoFisher Scientific.

### 2.2 Animals

Male *SD* rats (~300 g, 8 week) were purchased from DaRenFuChen Animal Co., Ltd. Rats were customized in cages in air‐conditioned animal room under a photoperiod schedule of 12‐hr light/12‐hr dark cycles at 25 ± 2°C. The rats were fed with normal food and tap water for 1 week before the experiments. Rats were fasted overnight before experiments with free access to tap water. All experiments with animals were carried out in accordance with the guidelines of Care and Use of Laboratory Animals published by China National Institute of Health.

### 2.3 Thrombolytic activity of NK‐01 in vivo

Fifteen rats were divided into negative control group, NK‐01 group, and positive control group. Rats were anesthetized by 3% Pelltobarbitalum Natricum (1 ml/kg body weight). Rats in negative control group and NK‐01 group were injected with 0.5 ml 0.9% NaCl and 0.5 ml NK‐01 (5,000 FU) into duodenal. Rats in positive control group were treated with urokinase (50,000 U) by tail vein injection. Four hours after injection, the blood of all rats was collected with tubes containing 3.2% sodium citrate solution (9:1, v/v). Blood was centrifuged at 3,000 g for 15 min at room temperature. Subsequently, the citrated plasma was used for analysis of thrombotic indexes (PT, APTT, TT, and FIB).

### 2.4 Preparation of proteomic samples

Twelve rats were divided into two groups: negative control group and NK‐01 group. Rats were anesthetized as mentioned above and were injected with 0.5 ml 0.9% NaCl and 0.5 ml NK‐01 (5,000 FU) into duodenal. Four hours after injection, the blood of all rats was collected with tubes containing 3.2% sodium citrate solution (9:1, v/v) and was centrifuged at 3,000 g for 15 min at room temperature.

A moderate amount of plasma, PBS, and protease inhibitors were added into 0.22‐μm membrane filter centrifuge tube with loaded packing (Thermo 191085305) and incubated for 1 hr at room temperature. Then, the tubes were centrifuged at 500 g for 1 min and the filtrates were collected. The packing was cleaned with PBS twice to collect the filtrates. All of the filtrates were concentrated by 3K ultrafiltration. The concentrated plasma samples were replaced with 8 M urea for three repeated times to appropriate volume. Finally, the protein concentrations of plasma samples were determined by bicinchoninic acid (BCA) method.

### 2.5 Protein digestion and peptides quantification

The plasma samples were digested with trypsin solution according to the standard procedure. The peptides were re‐suspended with 2% ACN, 0.1% TFA, and Sep‐Pak desalination. Then, each sample was vacuum dried. The peptides were finally quantified by Thermo Fisher Scientific (Thermo 23275).

### 2.6 Mass spectrometry analysis

Experiments were performed on a Q Exactive mass spectrometer coupled with Easy‐nLC 1,200. Peptide of each sample (0.5 μg/μl) was injected for nano LC‐MS/MS analysis. The peptide (2 μg) was loaded onto a C18‐reversed phase column (75 μm × 25 cm, Thermo) in buffer A (2% acetonitrile and 0.1% Formic acid) and separated with a linear gradient of buffer B (80% acetonitrile and 0.1% Formic acid) at a flow rate of 300 nl/min. In order to fully separate the peptides in plasma, the gradient elution program was set as follows: The concentration of (B) in the mobile phase was 0% at the initial, and then a linear increase from 3% to 6% (B) from 0 to 2 min, followed by a linear increase from 6% to 23% (B) from 2 to 105 min, and a linear increase from 23% to 29% (B) from 105 to 130 min, further a linear increase from 29% to 100% (B) from 130 to 149 min, finally 100% constant 100% (B) from 149 to 155 min. Electrospray ionization was carried out in the positive ionization mode. The electrospray voltage was 1.8 kV. Q Exactive mass spectrometer was operated in the data‐dependent mode to switch automatically between MS and MS/MS acquisition. Resolution for MS survey scan was set to 70 K for full‐scan MS spectra (m/z 350–1300) and 17.5 K for MS/MS scan. In all cases, one microscan was recorded using dynamic exclusion of 18 s. For MS/MS, normalized collision energy was set at 30 s.

### 2.7 Sequence database searching

MS/MS spectra were searched using PEAKS against Rattus norvegicus (29,978 entries) and the decoy database as the following parameters. The highest score for a given peptide mass (best match to that predicted in the database) was used to identify parent proteins. The parameters for protein searching were set as follows: tryptic digestion with up to two missed cleavages, carbamidomethylation of cysteines as fixed modification, and oxidation of methionines and protein N‐terminal acetylation as variable modifications. Peptide spectral matches were validated based on q‐values at a 1% false discovery rate.

### 2.8 Statistical analysis

All results were expressed as mean ± standard deviation ($\bar{X}\pm SD$), and statistical analysis was performed using Student's *t* test for comparison of two groups. In order to avoid problems such as the omission of identification results, the peptide error of the database search needs to be controlled within ± 10 ppm. To define the differentially expressed proteins, either a fold change (FC)>1.2 or a FC < 1/1.2 was set as the threshold. *p*‐value (<.05) was set as significant level.

## 3 RESULTS AND DISCUSSIONS

### 3.1 Thrombolytic activity of NK‐01 in vivo

The levels of prothrombin time (PT), activated partial thromboplastin time (APTT), thrombin time (TT), and fibrinogen (FIB) are important indicators to judge the pathological changes of coagulation system in the body. The levels of PT, APTT, and TT are decreased in the persons with thrombotic diseases. However, the level of FIB is usually increased. And it was reported that the level of PT could be decreased after treated with nattokinase (Kapoor, Harde, Jain, Panda, & Panda, 2015) And it was also reported that nattokinase not only possesses plasminogen activator activity, but also directly digests fibrin through limited proteolysis. Chien‐Hsun Hsia et al. conducted a clinical trial and showed that oral administration of nattokinase could be considered as a CVD nutraceutical by decreasing plasma levels of fibrinogen, factor VII, and factor VIII (Hsia et al., 2009). Yuko Kurosawa1 determined the quantitative effects of a single dose of nattokinase administration on coagulation/fibrinolysis parameters in healthy male subjects. They found that a single dose of NK administration could enhance fibrinolysis and anticoagulation via several different pathways simultaneously. (Chen et al., 2018).

**Figure 1:**
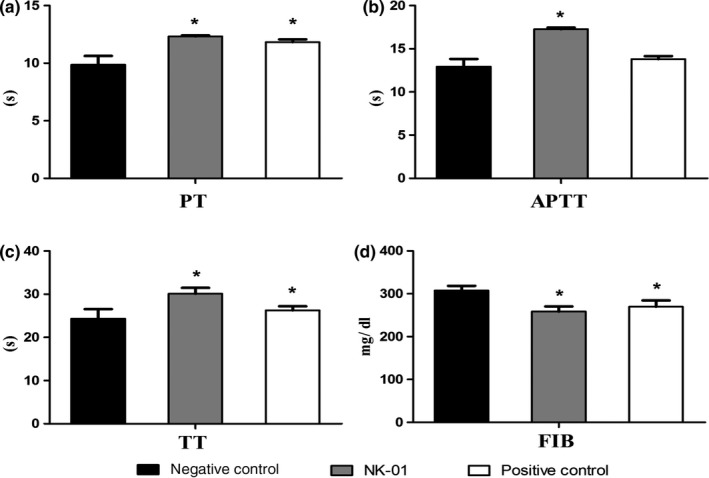
Changes of PT, APTT, TT, and FIB levels in rat. (a: PT, b: APTT, c: TT, d: FIB)

It was shown in Figure 1 that the levels of PT, APTT, and TT were significantly increased in the male rats treated with NK‐01 compared with the negative control group (*p* < .05). However, the level of FIB in the rats treated with NK‐01 was significantly decreased compared with the negative control group (*p* < .05). The result indicated that NK‐01 had potential in vivo antithrombotic activities.

### 3.2 Identification of proteins from quantitative proteomic analysis

**Figure 2:**
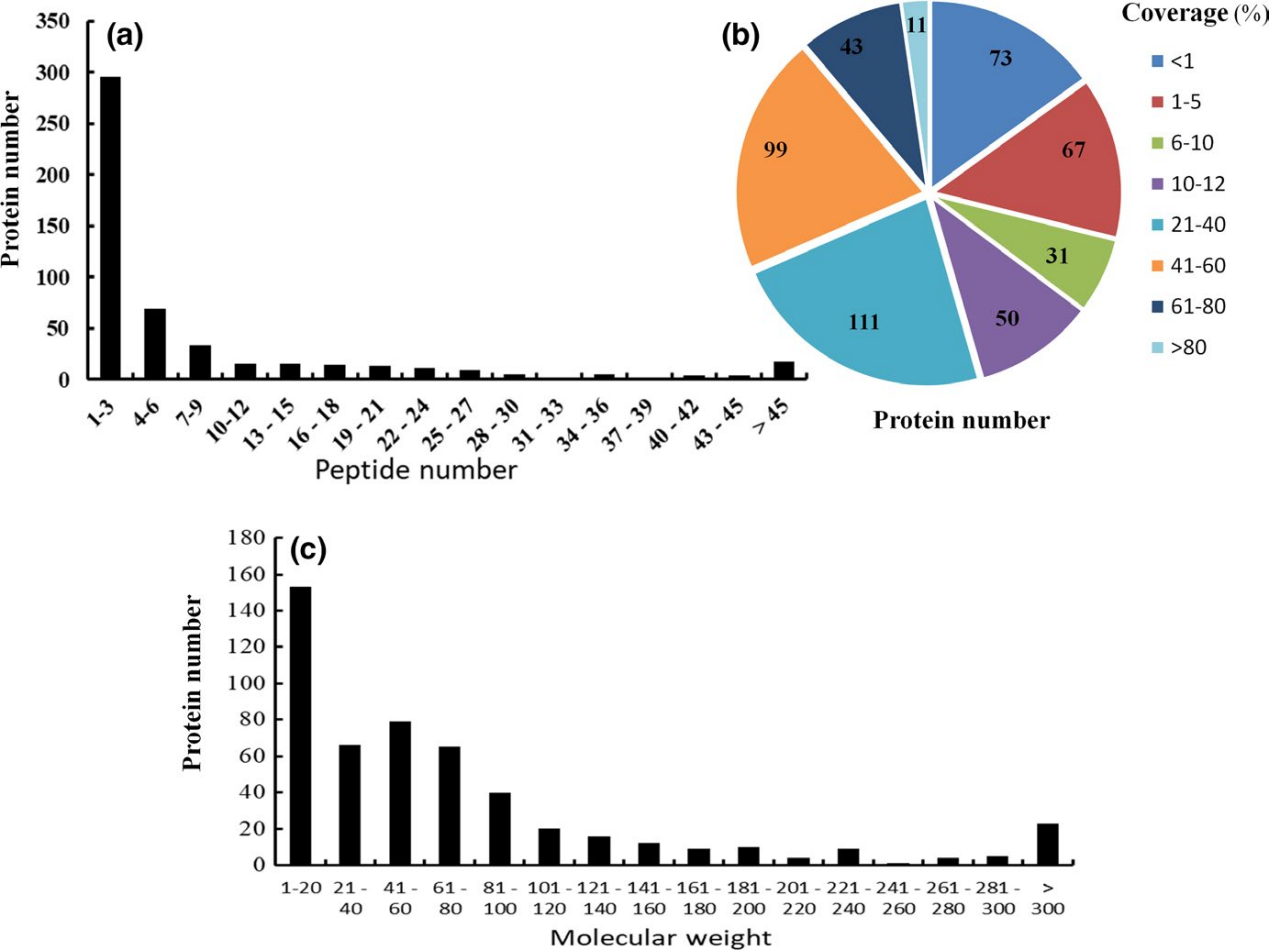
Distribution of peptide number, protein coverage, and protein molecular weight. (a) Distribution and number of peptides matched to proteins. (b) Coverage of proteins by identified peptides. (c) Protein molecular weight distribution

A total of 254,117 spectra were obtained from the LC‐MS/MS proteomic analysis. After data filtering, a total of 64,302 spectra were matched to 4,086 peptides, which were mapped to 665 proteins and 516 protein groups. Among all the identified proteins, approximately 57% of the proteins included 1–3 peptides and 43% of the proteins included at least 4 peptides (Figure 2a). In addition, the proteins were identified with high sequence coverage, 65% of proteins were identified with more than 10% of the sequence coverage, and 54% of proteins were identified with more than 20% of the sequence coverage (Figure 2b). Also, 42% of proteins were identified with the molecular weight less than 40 kDa and 58% of the proteins were identified with the molecular weight larger than 40 kDa (Figure 2c).

### 3.3 Analysis of differentially expressed proteins

Three biological replicate experiments were performed on the serum from two groups of rats. The experimental results showed that a total of 665 proteins were detected and 453 proteins could be quantified. Among them, there were 73 differentially expressed proteins including 49 up‐regulated (FC > 1.2, *p < .05) proteins and 24 down‐regulated proteins (FC < 0.83, *p < .05). The basic information for the up‐regulated proteins and down‐regulated proteins was shown in Table 1.

**Table 1:** List of relevant proteins identified as significantly differentially expressed in rats

Accession	FC (experiment/control)	Description
M0RE02	479.58	Uncharacterized protein
F1M0B7	16.01	Uncharacterized protein
M0RBL2	13.94	Uncharacterized protein
A0A0G2K458	10.58	Uncharacterized protein
D3ZEP5	9.92	Uncharacterized protein
O09171	9.58	Betaine—homocysteine S‐methyltransferase
M0R7M5	5.95	Uncharacterized protein
M0RC23	5.40	Uncharacterized protein
A0A0G2JY98	4.26	Uncharacterized protein
F1M7I8	4.05	Similar to Ig variable region light chain
M0RDL2	3.23	Uncharacterized protein
P01836	3.07	Ig kappa chain C region A allele
M0R816	2.99	Uncharacterized protein
A0A0G2KAH4	2.76	Dedicator of cytokinesis 6
D4ACR1	2.69	Uncharacterized protein
P23785	2.66	Granulins
D3ZWC1	2.57	Uncharacterized protein
P02770	2.56	Serum albumin
D3Z9Z7	2.54	Collagen beta(1‐O)galactosyltransferase 2
F1LXY6	2.50	Uncharacterized protein
P02761	2.34	Major urinary protein
A0A0G2JSH5	2.10	Serum albumin
M0R979	2.08	Uncharacterized protein
A0A0G2K0N6	2.04	Uncharacterized protein
M0RBP7	2.03	Uncharacterized protein
Q5M890	1.92	Apolipoprotein N
F1LZH0	1.86	Uncharacterized protein
D4A183	1.84	Similar to Vanin‐3 (Predicted)
Q9EPI1	1.81	Xylosyltransferase 1 (Fragment)
F1MAK3	1.81	Rho GTPase‐activating protein 32
Q6AYS3	1.69	Carboxypeptidase
D3ZP12	1.68	Zinc finger CCCH type containing 7 A (Predicted)
P04916	1.68	Retinol‐binding protein 4 OS = R
A0A0G2JX36	1.64	Uncharacterized protein
P55159	1.64	Serum paraoxonase/arylesterase 1
P53813	1.64	Vitamin K‐dependent protein S
M0RBX3	1.63	Uncharacterized protein
E9PSL7	1.63	Citron rho‐interacting serine/threonine kinase
M0R4C5	1.62	Uncharacterized protein
F1LVL2	1.59	Inducible T‐cell co‐stimulator ligand
Q6P734	1.55	Plasma protease C1 inhibitor
M0R628	1.49	Uncharacterized protein
A0A0G2K5X3	1.48	Uncharacterized protein
A0A0G2K980	1.43	Uncharacterized protein
D3ZCX6	1.32	RNA exonuclease 1 homolog
A0A0G2K151	1.29	Apolipoprotein E
A0A0G2K531	1.28	Glutathione peroxidase
P01015	1.26	Angiotensinogen
A0A0G2K4I9	1.25	Coagulation factor IX
A0A0G2JUY3	0.32	Uncharacterized protein
B1WC21	0.68	Fibulin−1
P01883	0.21	Ig delta chain C region (Fragment)
A0A0G2K9Z5	0.25	Uncharacterized protein
E9PTD7	0.18	Kin of IRRE‐like 3 (Drosophila)
A0A0H2UHM3	0.62	Haptoglobin
P02651	0.67	Apolipoprotein A‐IV
P08932	0.69	T‐kininogen 2
P01805	0.65	Ig heavy chain V region IR2
A0A0G2K2D9	0.38	Uncharacterized protein
A0A0G2K926	0.48	Alpha−1‐inhibitor III
A0A0G2JZL1	0.76	Uncharacterized protein
O88278	0.73	Cadherin EGF LAG seven‐pass G‐type receptor 3
P01855	0.49	Ig epsilon chain C region
P20766	0.63	Ig lambda−1 chain C region
A0A0G2JZN1	0.60	Uncharacterized protein
A0A140TAE6	0.82	Enoyl‐[acyl‐carrier‐protein] reductase mitochondrial
F7FIX1	0.38	Uncharacterized protein
F1M9L4	0.60	Cation‐transporting ATPase
D3ZFC6	0.79	Inter‐alpha‐trypsin inhibitor heavy chain family member 4
G3V7H3	0.58	Complement factor D
Q68FP1	0.72	Gelsolin
A0A1W2Q642	0.47	Uncharacterized protein
D3ZF92	0.67	Tumor necrosis factor receptor superfamily member 21

### 3.4 Influence of NK‐01 on key serum proteins involved in the thrombolysis and blood coagulation process

**Figure 3:**
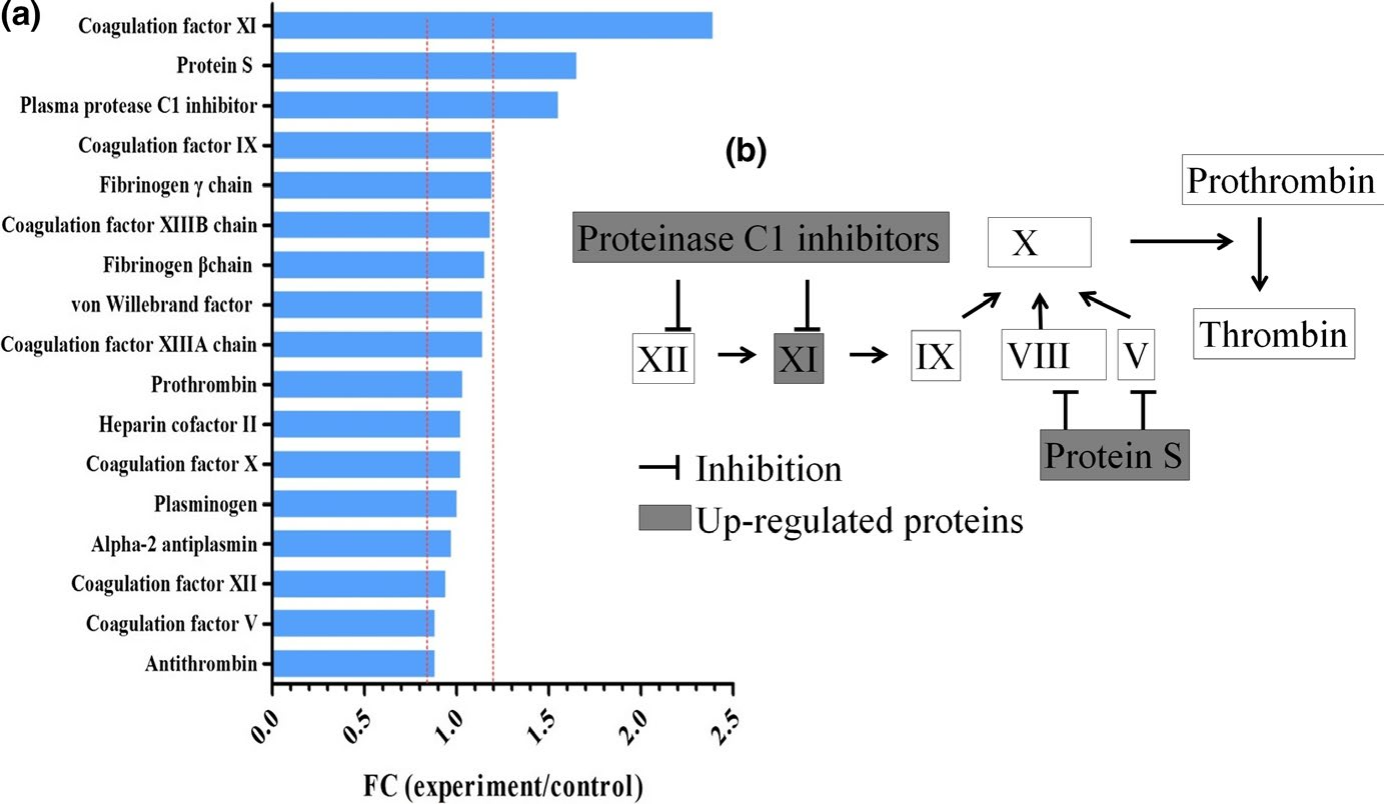
Identified proteins associated with fibrinolysis and coagulation system. (a) Showed the seventeen proteins associated with fibrinolysis and coagulation system. The dot lines mean the thresholds of the significantly differentially expressed proteins. (b) Showed the metabolic pathway which the proteinase C1 and protein S participate in and the function of them

As shown in Figure 3a, a total of 17 proteins that are associated with thrombolysis and coagulation processes, such as prothrombin, plasminogen, and coagulation factors, were detected. The proteinase C1 inhibitors and protein S were up‐regulated in normal rats after treatment with NK‐01, and others had no significant changes in normal rats after treated with high dose of NK‐01.

The roles of proteinase C1 inhibitors and protein S were illustrated in Figure 3b (Castoldi & Hackeng, 2008; Schurmann et al., 2014). Our data indicated that NK‐01 could affect the process of blood coagulation by influencing the activities of coagulation factors.

In addition, most of the 17 proteins that were detected had no significant fold change, suggesting that high dose of NK‐01 treatment cannot cause disorder in the thrombolytic process. Therefore, NK‐01 had good biosafety in terms of thrombolysis.

### 3.5 Influence of NK‐01 on key proteins involved in blood pressure regulation

**Figure 4:**
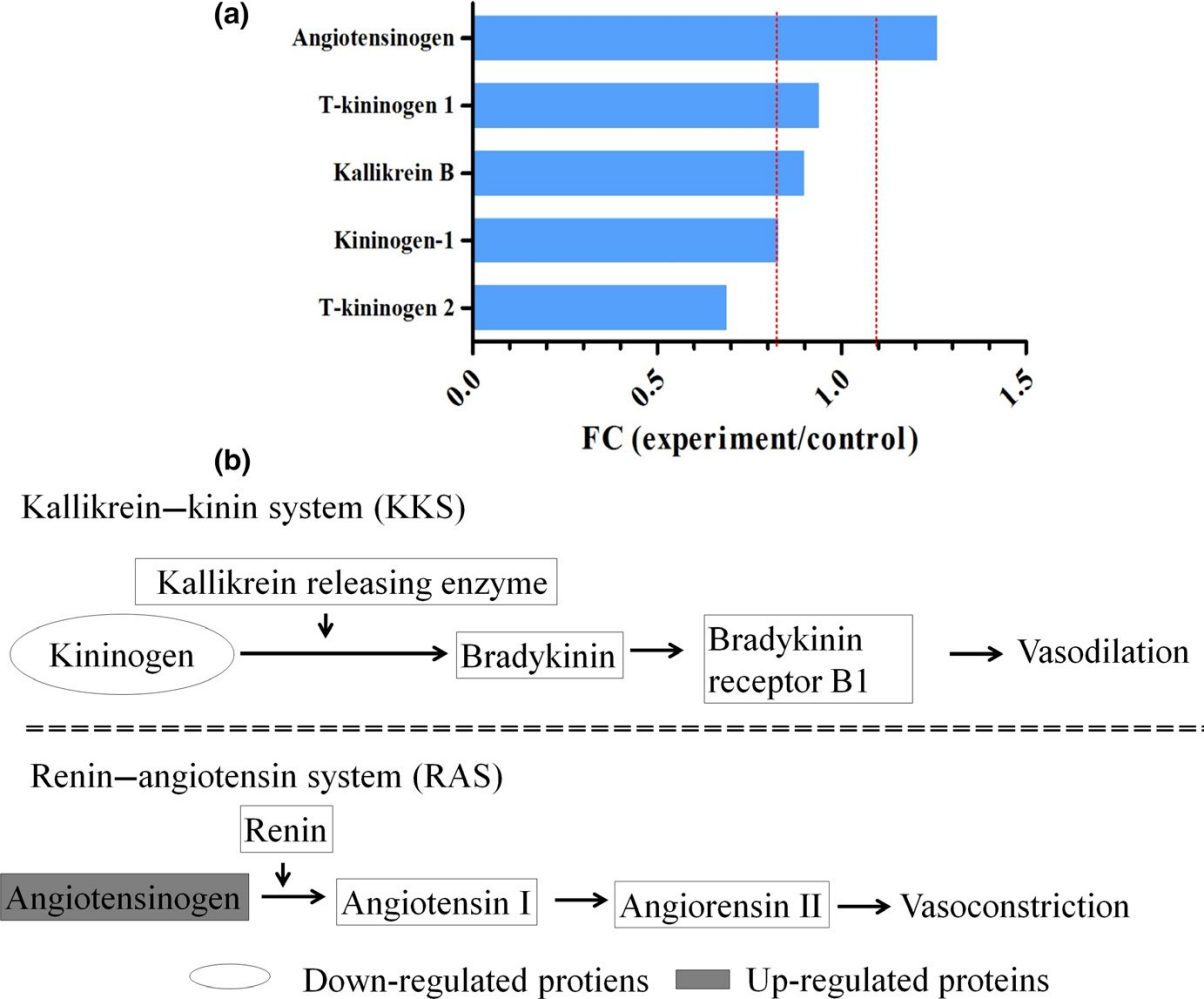
Identified proteins associated with regulation of blood pressure. (a) Showed the five proteins associated with **r**egulation of blood pressure. The dot lines mean the thresholds of the significantly differentially expressed proteins. (b) Showed metabolic pathway which the Angiotensinogen and T‐kininogen 2 participant and the function of them

Among the proteins detected, there were five proteins that were related to the regulation of blood pressure. The angiotensinogen was significantly up‐regulated, and the T‐kininogen 2 was significantly down‐regulated as shown in Figure 4a. Angiotensinogen and T‐kininogen 2 all played important roles in regulating blood pressure (Figure 4b).

Renin plays an important role in renin–angiotensin system (RAS) and could convert angiotensinogen to angiotensin I. Then, angiotensin I was converted to angiotensin II, which could raise the blood pressure directly (Lavoie & Sigmund, 2003). In addition, the kallikrein–kinin system (KKS) could lower blood pressure and negatively regulate RAS. In the KKS, kininogen is degraded into kinin, which is helpful for controlling the blood pressure (Regoli & Gobeil, 2017). It was reported that nattokinase could lower the blood pressure and had impact on the renin activity (Kim et al., 2008). Nattokinase could inhibit angiotensin I converting enzyme by a mixed type (the decrease in *V*max and the increase in *K*m value) to suppress blood pressure. (Murakami, Yamanaka, Ohnishi, Fukayama, & Yoshino, 2012). In our study, angiotensinogen was up‐regulated and the kininogen was down‐regulated in rats treated with NK‐01. This implied that nattokinase could inhibit the conversation of angiotensinogen and promote the degradation of kininogen. In this way, nattokinase could regulate the blood pressure.

### 3.6 Influence of NK‐01 on key proteins involved in blood lipid regulation

**Figure 5:**
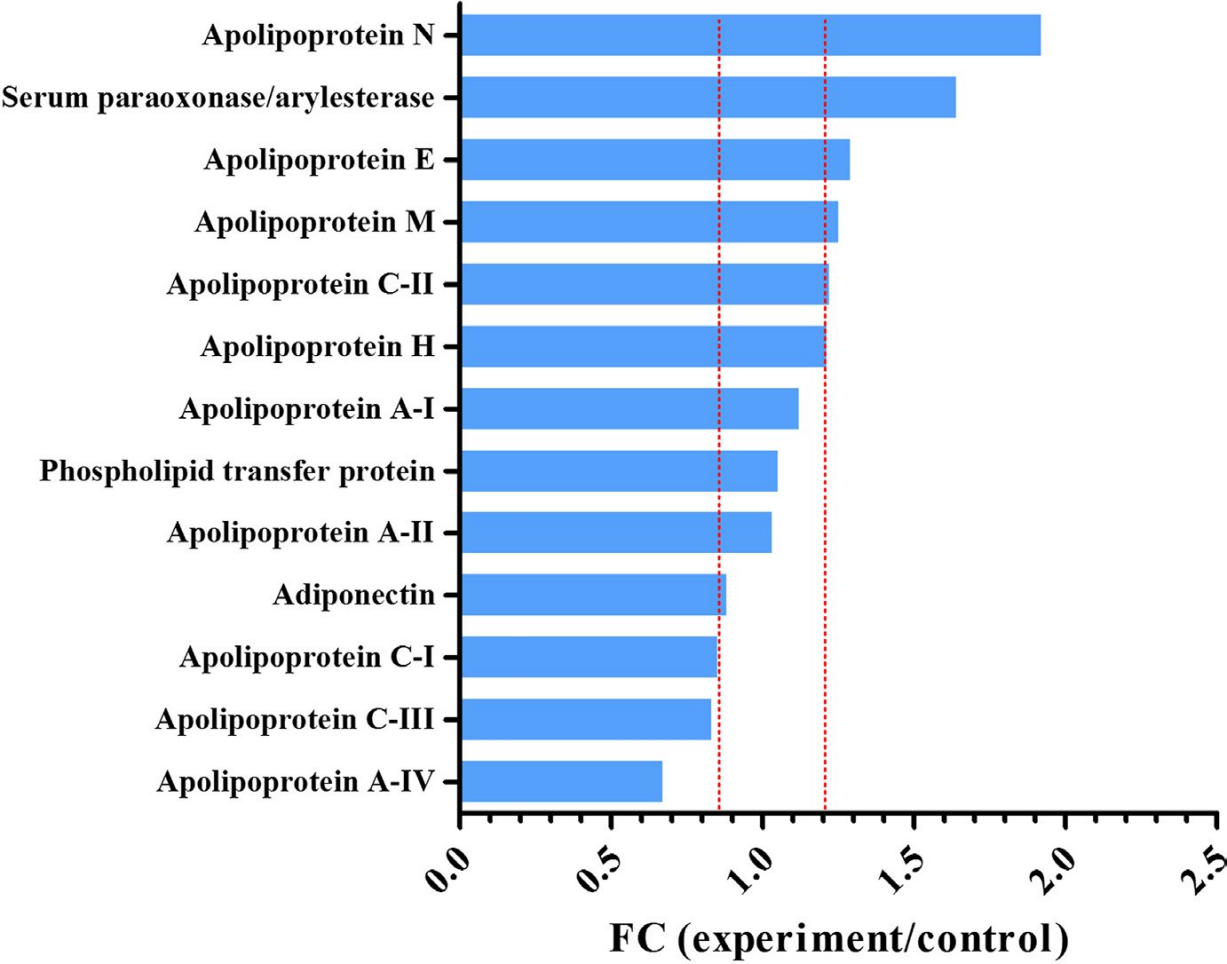
Identified proteins associated with regulation of blood lipids. This figure showed the thirteen proteins associated with regulation of lipids. The dot lines mean the thresholds of the significantly differentially expressed proteins

It was shown in Figure 5 that a total of 13 proteins that were associated with regulation of blood lipids were detected. Paraoxonase/arylesterase 1, apolipoprotein N, and apolipoprotein E were all up‐regulated, while apolipoprotein A‐IV was down‐regulated (Figure 5) Paraoxonase/arylesterase 1, a high‐density lipoprotein (HDL)‐associated enzyme, could inhibit the oxidation of LDL (Mackness, Quarck, Verreth, Mackness, & Holvoet, 2006). After treatment with NK‐01, the paraoxonase/arylesterase 1 was significantly up‐regulated in rats. Therefore, NK‐01 had the potential to prevent atherosclerosis. According to our results, the Apo E was up‐regulated and the Apo A‐IV was down‐regulated in normal rats after treatment with NK‐01. The possible reason might be because that the rats were fasted overnight and need more LDL to maintain normal metabolic.

## CONFLICT OF INTEREST

The authors declare that they do not have any conflict of interest. All the authors approve the submission of this manuscript and will take responsibility for any conflict of interest.

## ETHICAL APPROVAL

This study was approved by the Institutional Review Board of Ocean University of China, and the study's protocols and procedures were ethically reviewed and approved by China National Institute of Health.
